# The Vienna morphological Achilles tendon score—VIMATS: Description, reproducibility and initial clinical results

**DOI:** 10.1007/s00508-021-01863-6

**Published:** 2021-06-03

**Authors:** Sebastian Apprich, Arastoo Nia, Markus M. Schreiner, Klaus Friedrich, Reinhard Windhager, Siegfried Trattnig

**Affiliations:** 1 Department of Orthopaedic Surgery, Medical University of Vienna, Währinger Straße 18–20, 1090 Vienna, Austria; 2 Department of Trauma Surgery, Medical University of Vienna, Vienna, Austria; 3 Department of Radiology, Medical University of Vienna, Vienna, Austria; 4 High Field MR Centre, Department of Biomedical Imaging and Image-Guided Therapy, Medical University of Vienna, Vienna, Austria

**Keywords:** Achilles tendon, Tendinopathy, Magnetic resonance imaging, Radiological assessment, Scoring

## Abstract

**Objective:**

The purpose was to introduce the Vienna morphological Achilles tendon score (VIMATS), to evaluate its reproducibility and to assess its clinical application.

**Methods:**

In 38 patients a total number of 40 painful ATs and 20 volunteers were examined on a 3T magnetic resonance imaging (MRI) scanner using a standard MRI protocol. In 20 patients clinical scoring according to the Achilles tendon rupture score was available. Two observers independently assessed the thickness, continuity, signal intensity, and associated pathologies of the Achilles tendon (AT) according to the newly created VIMATS. Intraobserver and interobserver agreements were calculated and the clinical application of the VIMATS regarding its potential to differentiate between patients and volunteers was tested.

**Results:**

An analysis of the Intraclass correlation coefficient (ICC) yielded an excellent intraobserver (ICC 0.925) and interobserver agreement (ICC 0.946) for the total VIMAT score. A significant difference in total VIMATS was found between patients (47.6 ± StD 21.1 points) and volunteers (91.5 ± SD 10.9 points; *p* < 0.01) as well as a moderate correlation between morphological and clinical scoring (Pearson correlation 0.644).

**Conclusion:**

The VIMAT score is the first MRI score for the semiquantitative morphological evaluation of AT injuries and was shown to be an easy, fast and reproducible tool for assessing injuries of the AT.

## Introduction

The Achilles tendon (AT) is the strongest tendon in the human body [1] and is affected by various types of extrinsic (traumatic and overuse conditions) and intrinsic (gender, age, genetics) causes [2]. A recent study [3] found that Achilles tendinopathy is one of the most common overuse injury in young active adults. Furthermore, spontaneous rupture has become more common recently due to an increase in the sporting activity by the middle-aged population [4, 5].

In cases of acute AT ruptures, physical examination has shown similar sensitivity in diagnostics to magnetic resonance imaging (MRI); however, in cases of ambiguous presentations and subacute or chronic injuries of the AT, MRI still represents the gold standard in diagnostics [6]. A MRI can provide important information about the pathological state of the AT as well as associated pathologies. Furthermore, in a study by Khan et al. grading of the AT by baseline MRI appearance was associated with clinical outcome at the 12-month follow-up [7].

However, to date, no MRI score exists that utilizes the outstanding soft tissue contrast of MRI to semiquantify AT injuries by the morphological appearance in a standardized manner. Instead, most recent studies have attempted to align irregularly applied nomenclatures for different AT pathologies, such as achillodynia, tendinopathy and tendinosis [2, 8]. Therefore, we aimed to develop a new comprehensive MRI score that includes the relevant parameters for clinical and radiological physicians in a standardized, semiquantitative manner (meaning that different qualitative and quantitative parameters are scaled and contribute to an overall score of 0–100 points), comparable e.g. to the MOCART 2.0 score [25]. Semiquantitative MRI-based assessments may enable identification of AT tissue pathologies that are relevant to important clinical and structural endpoints.

Therefore, the purpose of this study was to develop and introduce the MRI-based, semiquantitative Vienna morphological Achilles tendon score (VIMATS), to evaluate its reproducibility in patients with AT injuries, to assess its potential to differentiate between patients and healthy volunteers and to assess its correlation with a clinical score for the first time.

## Material and methods

### Study cohort

Inclusion criteria for all patients were a minimum age of 18 years and a history of a painful AT for at least of 1 month and still painful at the time of the MRI scan. For this baseline study we excluded all patients with previous surgery of the AT as well as subjects with contraindications for MRI. No patients with acute AT injuries were included. Finally, 38 consecutive patients (mean age 49.9 ± SD 12.3 years, range 25–81 years, 10 females, 30 males, 27 right, 13 left) with a total number of 40 painful ATs (in 2 patients both sides were examined) were enrolled in this prospective study within 1 year. In 20 patients (patients were interviewed on telephone after MRI examination), clinical scoring according to the Achilles tendon total rupture score (ATRS, 0–100 points) was available [9]. The ATRS is a patient-reported instrument for measuring symptoms and physical activity related to the AT including 10 items of functional and daily activities each accounting for 0–10 points.

Additionally, 20 age (*p* = 0.07) and gender matched (*p* = 0.23) healthy volunteers with no history of AT pain or injury and an ATRS of 100 points were included in this study as a control cohort (mean age 42.8 ± SD 14.7 years, range 25–68 years, 8 females, 12 males, 10 left, 10 right).

### MRI protocol

The MRI examinations were performed on a 3.0 T whole-body Magnetom TimTrio scanner (Siemens Healthcare, Erlangen, Germany), using a gradient strength of 40 mT/m and an 8‑channel coil (In vivo, Gainesville, FL, USA). The standard MRI protocol [10] was identical for all examinations and consisted of a set of localizers and three morphological MRI sequences: 1) a sagittal fat-suppressed (fs) proton density-weighted (PD-w) turbo spin echo (TSE) sequence (TR 3970 ms, TE 26 ms, FoV 220 × 220 mm, TA 3:55 min); 2) a sagittal T1‑w spin echo (SE) sequence (TR 724 ms, TE 11 ms, FoV 220 × 220 mm, TA 3:22 min) and 3) an axial T2‑w TSE sequence (TR 6720 ms, TE 100 ms, FoV 170 × 170mm, TA 3:22 min). Total imaging time including positioning and registration of the patient was around 15 min.

### Image analysis according to the VIMAT score

The evaluation was performed on a picture archiving and communication system (PACS) workstation. Two observers (S.T. a senior musculoskeletal radiologist and S.A. an orthopedic surgeon with a special interest in musculo sceletal-MRI) independently assessed the MRI images according to the newly created VIMATS. The definition of the particular variables of the VIMAT score as well as their scaling and weighting within the total score was based on a discussion between orthopedic and radiologic coauthors. Special attention was paid to the requirement that the new score compromises the essential radiological variables with clinical relevance in a user-friendly manner that makes it applicable also for physicians who are not experts in the field of MRI.

All patient and volunteer data were anonymized and the evaluation was performed in random order. Observer 1 (S.T.) repeated the evaluation for patient data after a time interval of 3 months for intraobserver reliability assessment.

**Fig. 1:**
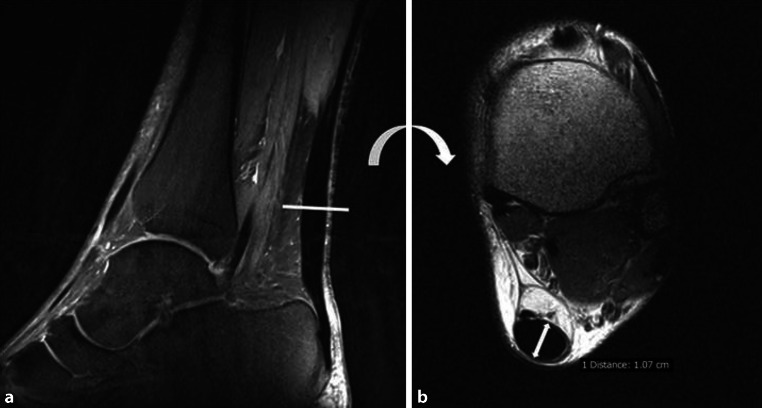
Correct measurement of the Achilles tendon thickness—measured distance from the anterior to the posterior margin of the tendon

**Fig. 2:**
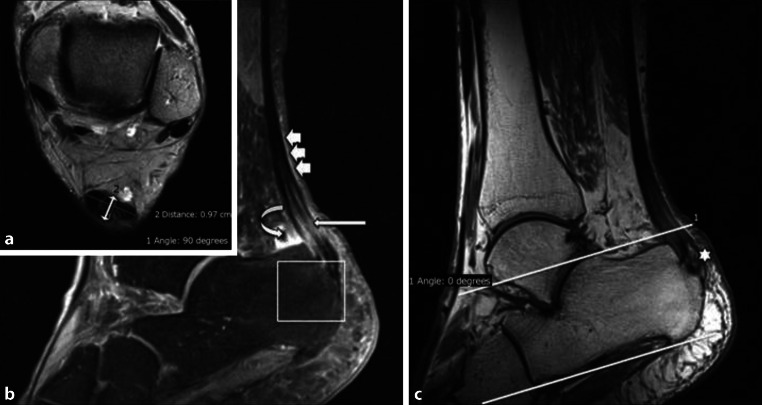
Case example of a 51-year-old female patient with pain in the AT for 2 months. The maximum thickness of the AT from anterior to posterior, perpendicular to the coronal axis of the AT, on the axial T2‑w image (**a**; *double-headed arrow*), was 9.7 mm (20 points). Furthermore, the patient presented with a partial tear (10 points) and fluid-like signal intensity (0 points; *long arrow*) on the sagittal PD‑w TSE image (**b**). Associated pathologies (20 points): no Haglund exostosis (minus 0 points; *parallel lines* (**c**)), no calcaneal bone marrow edema or cysts (**b**) but an ossification at the tendon insertion area (–5 points; *star*) on the T1‑m sagittal image (**c**); no edema of Kager’s fat pad but slight signal increase along the AT indicating a peritendinitis (**b**) (–5 points; *small arrows*); positive retrocalcaneal bursitis (**b**) (–5 points; *curved arrow*). Total VIMAT score = 35 points

Standardized assessment of the MRI images was performed according to the individual variables of the newly defined VIMATS:

1. Thickness
  The thickness of the AT is assessed on axial T2-weighted images, coregistered side by side with sagittal fs PD‑w TSE images, to ensure that the level of greatest thickness is measured. The maximum distance from the anterior to the posterior margin of the tendon in relation to the spiral orientation of the AT on the axial T2‑w images (no strict anterior-posterior orientation) is measured in millimeters. (Fig. 1). Subsequently, the thickness of the AT is divided into 4 groups from ≤7 mm (30 points) to ≥13 mm (0 points).
2. Continuity
  The continuity of the AT is determined on the sagittal fs PD‑w TSE images, coregistered side by side with axial T2-weighted images as normal (30 points; no discontinuity in the axial or sagittal direction), interstitial tear (20 points; irregular and interrupted linear areas of increased signal intensity parallel to the long axis of the AT on a minimum of two consecutive slices) [1]; partial tear (10 points; characterized by heterogeneous high T2-weighted signal intensity, and incomplete interruption of the tendon fibers with partial retraction, or a corkscrew appearance), or complete tear (0 points; characterized by fluid-filled tendinous gaps with retracted torn tendon fibers [1, 11]).
3. Signal intensity
  The signal intensity is evaluated according to the following subgroups on sagittal fs PD‑w TSE images and axial T2-weighted images: isointense signal (20 points; consistent low signal throughout the whole course of the AT); hyperintense signal (10 points; areas of increased but not fluid-like signal intensities on a minimum of two consecutive slices); fluid-like signal (0 points; areas of the same signal intensity as fluid on a minimum of two consecutive slices).
4. Associated pathologies
  For each observed pathology from a certain subgroup, 5 points are subtracted from the initial20 points. If none of the following associated pathologies are found, the full 20 points contribute to the total VIMAT score.
  - Haglund exostosis (‑5 points)
    The diagnosis of an enlarged calcaneal tuberosity (Haglund exostosis) on sagittal T1‑w SE images is made by drawing parallel pitch lines on the upper and lower aspects of the calcaneus on sagittal images (Fig. 2c) as described in the literature. In cases of Haglund’s disease, a portion of the tuberosity is seen above the upper pitch line [1, 12].
  - Tendon insertion area abnormalities (‑5 points)
    Alterations at the insertion site of the AT include enthesiophytes and ossification (sagittal T1‑w SE images), as well as cystic alterations and bone marrow edema (on sagittal fs PD‑w TSE and axial T2 images) at the transition from the insertion zone to the calcaneal bone.
  - Peritendinitis or edema of Kager’s fat pad (‑5 points)
    On T2‑w and PD‑w MRI sequences, peritendinitis appears as a linear or reticular high signal area alongside the deep surface as well as the subcutaneous surface of the tendon, representing an area of edema, thickened peritendinous tissue with fibrinous exudate, or increased vascularity [2, 10]. Edema of Kager’s fat pad is present if high signal is seen within the fat pad on the sagittal fs PD‑w TSE images.
  - Retrocalcaneal or subcutaneous bursitis (‑5 points)
    A bursitis of the retrocalcaneal bursa is defined as a fluid-like signal increase with a diameter more than 6 mm from superior to inferior and more than 3 mm from anterior to posterior on sagittal PD‑w images [13]. The subcutaneous calcaneal bursitis is directly subcutaneous and posterolateral to the bony insertion, and appears as a hyperintense structure on T2‑w and PD‑w MRI [1, 14]

The detailed distribution of points for the different variables of the VIMATS is shown in Table 1. The maximum score is 100 points.

**Table 1:** Individual variables of the VIMATS and its respective components contributing to a maximum score of 100 points

Variable	Points
*1. Thickness (AP orientation)*	
≤7 mm	30
7.1–10 mm	20
10.1–13 mm	10
≥13.1 mm	0
*2. Continuity*	
Normal	30
Interstitial tear	20
Partial tear	10
Complete tear	0
*3. Signal intensity*	
Isointense	20
Hyperintense	10
Fluid-like	0
*4.* Associated* pathologies*	
None	20
Haglund exostosis	−5
Tendon insertion area abnormalities (enthesiophytes, ossifications, cysts, calcaneal bone marrow edema)	−5
Peritendinitis/edema of Kager’s fat pad	−5
Retrocalcaneal and/or subcutaneous bursitis	−5
Maximum possible score	100

The study protocol was approved by the institutional ethics committee.

### Statistics

All statistical analyses were performed using SPSS for Mac version 24.0 (SPSS Institute, Chicago, IL, USA). Metric variables, such as the overall VIMAT score, are displayed using mean values and standard deviations (SD). Interobserver and intraobserver agreement for the total VIMATS and its individual variables was evaluated using intraclass correlation coefficients (ICC) and weighted (w) kappa statistics (evaluated according to the criteria defined by Landis and Koch [15]). Furthermore, the correlation between morphological scoring (VIMATS) and clinical scoring (ATRS) was tested using the Pearson and Spearman rank correlation.

Differences between patients and volunteers were evaluated using unpaired T‑test, Mann-Whitney U‑test and χ^2^-Test.

## Results

### Descriptive data

The mean VIMATS in patients for observer 1 was 47.6 ± standard deviation (SD) 21.1 points (range 15–100 points) for the first and 46.6 ± SD 23.2 points (range 5–100 points) for the second evaluation. For observer 2, a nearly identical mean VIMATS was found (47.2 ± SD 23.3 points, range 10–100 points). In volunteers, the mean VIMATS was 91.5 ± SD 10.9 points (range 70–100 points).

An illustrative example of an entire assessment of an AT according to the VIMATS is presented in Fig. 2.

Descriptive data about the analysis of both reviewers for the individual variables are given in Table 2.

**Table 2:** Descriptive data for the individual variables of the VIMAT score according to the assessment of the two observers in patients and volunteers. Percentages are displayed in parentheses

	Thickness				Continuity				Signal intensity		
	≤7 mm	7.1–10 mm	10.1–13 mm	≥13.1 mm	Normal	Interstitial tear	Partial tear	Complete tear	Isointense	Hyperintense	Fluid-like
Observer 1/1	4 (10)	11 (27.5)	14 (35)	11 (27.5)	13 (32.5)	12 (30)	12 (30)	3 (7.5)	2 (5)	19 (47.5)	19 (47.5)
Observer 1/2	4 (10)	10 (25)	13 (32.5)	13 (32.5)	8 (20)	14 (35)	15 (37.5)	3 (7.5)	5 (17.5)	20 (50)	15 (37.5)
Observer 2	4 (10)	13 (32.5)	12 (30)	11 (27.5)	11 (27.5)	8 (20)	18 (45)	3 (7.5)	4 (10)	21 (52.5)	15 (37.5)
Volunteers	17 (85)	3 (15)	0 (0)	0 (0)	16 (80)	4 (20)	0 (0)	0 (0)	13 (65)	7 (35)	0 (0)
–	**Associated pathologies**										
	*Haglund exostosis*		*Tendon insertion area abnormalities*			*Peritendinitis/edema of Kager’s fat pad*			*Bursitis*		
	Yes	No	Yes	No		Yes	No		Yes	No	
Observer 1/1	6 (15)	34 (85)	21 (52.5)	19 (47.5)		31 (77.5)	9 (22.5)		13 (32.5)	27 (67.5)	
Observer 1/2	5 (12.5)	35 (8.5)	21 (52.5)	19 (47.5)		30 (75)	10 (25)		13 (32.5)	27 (67.5)	
Observer 2	6 (15)	34 (85)	23 (57.5)	17 (42.5)		30 (75)	10 (25)		15 (37.5)	25 (62.5)	
Volunteers	2 (10)	18 (90)	1 (5)	19 95)		3 (15)	17 (85)		0 (0)	20 (100)	

### Reliability

The ICC yielded an excellent intraobserver (ICC 0.925) and interobserver agreement (ICC 0.946) for the total VIMATS.

Concerning the intraobserver agreement for individual variables, kappa values ranged from 0.517 for peritendinitis to 0.886 for the assessment of bursitis.

Excellent interobserver agreement was found for the variable thickness (kappa value 0.931); worst, but still moderate, interobserver agreement was found for the variable peritendinitis and edema of Kager’s fat pad (kappa value 0.655) (Table 3).

**Table 3:** Collective data for interobserver and intraobserver agreement for individual variables and total VIMAT score

	Thickness (mm)^a^	Thickness^b^	Continuity^b^	Signal^b^	Associated pathologies				Total VIMAT-Score^1^
					Haglund exostosis^b^	Tendon insertion area abnormalities^b^	Peritendinitis/edema of Kager’s fat pad^b^	Bursitis^b^	
Intraobserver Agreement	0.945 (0,898–0.970)	0.826	0.649	0.568	0.684	0.799	0.517	0.886	0.925 (0.863–0.960)
Interobserver Agreement	0.968 (0.941–0.983)	0.931	0.718	0.780	1	0.698	0.655	0.890	0.946 (0.901–0.971)

^a^intraclass correlation coefficient (ICC) (95% confidence internal, CI)

^b^weighted hierarchical kappa

### Correlation with clinical scoring

**Fig. 3:**
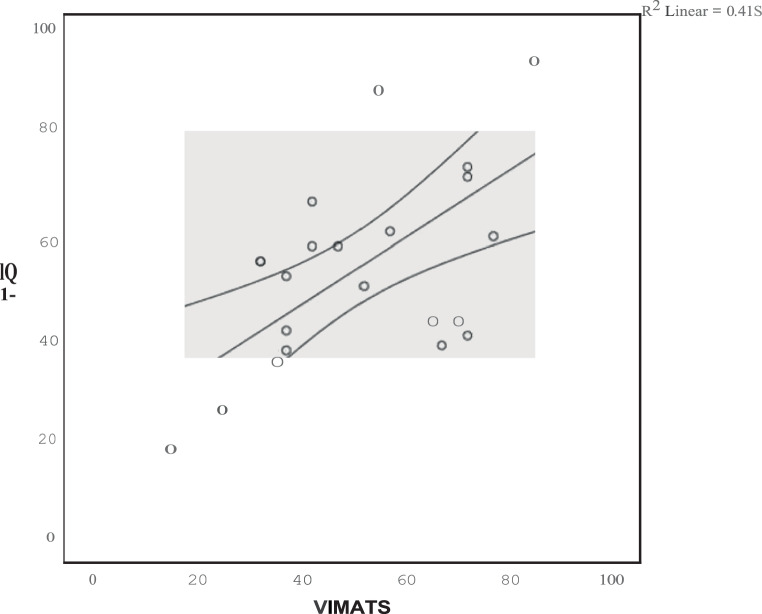
Correlation between clinical scoring according to the Achilles tendon rupture score and the total score of the Vienna morphological Achilles tendon score (0.644) - X Axis: VIMATS points, Y axis: interquartile

Pearson correlation between ATRS and VIMATS yielded a moderate correlation of 0.644 (Fig. 3). Highest correlation between clinical scoring and the individual variable of the VIMATS was found for thickness (−0.789), signal intensity (−0.784) and continuity (−0.677), whereas the correlation with associated pathologies ranged between −0.609 for tendon insertion area abnormalities and −0.201 for Haglund exostosis.

### Patients vs. volunteers

The total VIMATS (*p* < 0.01) as well as the distribution of the particular variables (*p* ≤ 0.04) differed significantly between patients and volunteers. Only the incidence of Haglund exostosis was not increased in the patient cohort in comparison to volunteers (*p* = 0.591).

## Discussion

This study introduces the newly developed semiquantitative, MRI-based Vienna morphological Achilles tendon score (VIMATS) and describes the initial experience in assessing patients with AT pain.

The intent of this study was to develop and provide an easy and reproducible semiquantitative assessment tool for AT disorders that would also find widespread acceptance among physicians who are not experts in the field of MRI and can be used in daily clinical routine. Therefore, only morphological standard sequences were used for the evaluation of the score. Furthermore, we kept the overall scan time of the MRI protocol to about 15 min, which is feasible in a clinical or clinical trial setting.

Schweitzer and Karasick proposed a clinical radiological tendinopathy classification of 7 relevant main groups including a total of 11 subgroups with different imaging characteristics [1]. The present score does not try to classify a specific pathology into a nomenclature. Rather, by combining several morphological characteristics, injuries of the AT can be semiquantitatively scored from 0 to 100 points in a standardized manner. This might help in the future to easily compare the morphological results of various treatment procedures.

Based on our new VIMATS excellent interobserver (0.946) and intraobserver agreement (0.925) was found. As explained before, the score consists of four different parameters: (1) thickness, (2) continuity, (3) signal and (4) associated pathologies, with four subgroups of possible associated pathologies.

Normal tendon thickness is described to be less than 7 mm in healthy asymptomatic volunteers [10, 16]. With ongoing degeneration of the AT, swelling of the tendon occurs. Therefore, the variable thickness was chosen as a substantial parameter of the VIMAT score, counting for 30% of the maximum score. Some authors suggested measuring the volume of the AT [17]; however, this requires appropriate software tools.

The continuity of the tendon is the most important factor for the functionality of the AT, and therefore, we weighted this variable also with possible 30 points within the VIMAT score. The most typical location for a rupture of the AT is 2–6 cm proximal of the calcaneal insertion, because there is a hypovascular zone with reduced nutrition of the tissue [18]. It is important for the functionality as well as the clinical outcome if there is a complete or a partial tear. A possible pitfall when assessing interstitial tears of the AT is the normal fascicular anatomy or small intratendinous vessels of the AT. These may be visible as a single hyperintense line and can mimic an interstitial tear but do not show fluid-like signal intensity and do not have a pathological value [1, 10].

The signal intensity counts for another 20% (20 points) of the total score. Due to the very short relaxation times of the tendon fibers, the healthy AT should be low of signal and almost dark; however, besides the fact of free water within the tendon as well as change in fiber orientation (magic angle effect) as a cause of rupture, a variety of tendon degenerations (which can be hypoxic, hyaline, myxoid, fibrinoid, or fatty [2, 19]) lead to an increase in T2 relaxation times and are seen as a precursor for a weakening of the AT structure. The relative high percentage of 35% of asymptomatic tendons with hyperintense intratendinous signal intensity in the control group might be due to neovascularization and is consistent with the literature [20].

The variables thickness, continuity and signal intensity account together for 80% of the total VIMATS. This seems to be justified in consideration of the fact that these variables showed the highest correlation with clinical scoring.

Furthermore, the VIMAT score takes different associated pathologies into account the presence of which is a cause for or expression of inflammation or degenerative processes, and have a negative impact on the status of the AT, and therefore, on the total score of the individual VIMATS (up to minus 20 points).

The Haglund exostosis is also known as “pump bump” [14] and ends in a circle of injury, response to injury and reinjury [1]. As a result of chronic irritation, the calcaneal tuberosity may enlarge, which further irritates the retro-Achilles bursa and the AT itself, which again leads to an irritation of the calcaneal tuberosity. Therefore, it may also be associated with retrocalcaneal bursitis or insertional tendinitis [21]; however, in our small cohort of patients and volunteers no difference was found in the incidence of the Haglund deformity.

The insertional tendonitis is common in runners and frequently leads to the development of calcifications, bone spurs and bone cysts in the tendon insertion at the calcaneus [1, 14], often seen as the source for back heel pain. Likewise, the calcaneal bone marrow edema as a result of direct trauma or chronic failure load.

Paratendonitis and peritendinitis are frequently used synonymously and refer to the inflammatory change of the paratendon. With an acute peritendinitis, the tendon maintains its normal size and shape, whereas a chronic peritendinitis leads to a thickening of the tendon [14]. Due to the increased fluid content in the inflamed tissue, a peripheral signal intensity increase can be seen on T2-weighted images.

In the case of degenerative AT changes, edema of Karger’s fat pad and concomitant retrocalcaneal bursitis are often present. A subcutaneous bursitis often occurs and its presence usually indicates local trauma or inflammation.

Regarding the clinical value of the presented score, we were able to initially show that the individual variables (with exception of the incidence of the Haglund exostosis) of the VIMATS as well as the total VIMATS itself differed significantly between patients and healthy volunteers. Furthermore, the initial results for correlation with clinical scoring yielded moderate results; however, to clarify the clinical value and the accuracy of the newly created VIMATS, some future work has to be done. Numerically larger as well as more accurately defined patient cohorts (for example classification of AT pathologies according to the classification proposed by Schweitzer and Karasick) need to be investigated using the VIMATS. Furthermore, follow-up studies using the VIMATS on different treatment strategies will strengthen its clinical implication. Several studies have demonstrated the feasibility of different new quantitative MRI methods for the evaluation of the biochemical composition and ultrastructure of the AT [22, 23]. Correlation of the VIMAT score with these new quantitative MRI techniques will further strengthen the clinical value of the VIMAT score in the assessment of AT pathologies.

A limiting factor of this study is the comparatively small number of patients enrolled. To confirm the validity of the assessed parameters and the VIMAT score itself, correlation with a gold standard, such as intraoperative reports or histology would be necessary. Clinical scoring using the ATRS was only available in 20 patients because this was done retrospectively in telephone interviews, which is a major limitation of this study. Furthermore, for this initial study only preoperative patients were included. Of course, the usability of this score in postoperative patients needs to be proven in future studies. Since most of the available MRI scanners have field strengths lower than 3.0 T, a MRI scanner with a field strength of 1.5 T, for example, may have a negative impact on the validity and reproducibility of the VIMATS; however, in a more recent study, the MRI evaluation of joints and tendons of the hindfoot did not reveal any significant differences between low-field and high-field MRI [24]. Furthermore, no contrast agent was added to the MRI protocol. Contrast enhancement might have increased the diagnostic accuracy, especially when an inflammation process is present; however, standard administration of contrast agent would reduce the clinical applicability of this score. Nevertheless, if an inflammation process is suspected decision for administration of contrast agent can be done in each case individually.

In conclusion this study demonstrated that the VIMATS is a straightforward (applicable also for physicians who are not experts in the field of MRI imaging), fast (short and cost-efficient MRI protocol), and reproducible MRI score which was able to distinguish between patients and volunteers and showed moderate correlation with clinical scoring. Further evaluation of the clinical validity of the presented MRI scoring system should be the subject of additional future prospective studies. This might in the future provide physicians with a powerful tool to longitudinally monitor patients before and after surgical or conservative treatment and might help, e.g. to predict patients who are at risk of rupture or rerupture of the Achilles tendon.
